# Preoperative Prognostic Nutritional Index and Neutrophil-to-Lymphocyte Ratio Predict Survival Outcomes of Patients With Hepatocellular Carcinoma After Curative Resection

**DOI:** 10.3389/fonc.2021.823054

**Published:** 2022-01-28

**Authors:** Zhen Qu, Yun-jie Lu, Jia-Wei Feng, Yu-xiang Chen, Long-qing Shi, Jing Chen, Navin Rambaran, Yun-Fei Duan, Xiao-zhou He

**Affiliations:** ^1^ The Third Affiliated Hospital of Soochow University, Changzhou First People’s Hospital, Changzhou, China; ^2^ Department of General Surgery, Georgetown Hospital Complex, Georgetown, Guyana

**Keywords:** hepatocellular carcinoma, prognostic nutrition index, neutrophil-to-lymphocyte ratio, survival, prognostic factors

## Abstract

Increasing evidence indicates that preoperative prognostic indices can serve as independent predictors of survival in patients with cancer. However, the applicability of these indices in patients with hepatocellular carcinoma (HCC) is controversial. This study aims to investigate the prognostic value of these indices in patients with HCC after curative hepatectomy. We retrospectively analyzed the data of 215 patients who underwent curative resection for HCC. Prognostic indices including prognostic nutritional index (PNI) and neutrophil-to-lymphocyte ratio (NLR) were evaluated by comparing by the area under the curve (AUC). Univariate analysis and multivariate analysis were performed to identify independent prognostic factors. Additionally, risk factors were combined to predict the survival of patients. We found that serum albumin concentration, tumor diameter, tumor stage, degree of differentiation, PNI, and NLR were independent prognostic factors for overall survival (OS). Vascular invasion, tumor stage, degree of differentiation, and PNI were independent prognostic factors for recurrence-free survival (RFS). The cutoff value of the PNI and NLR was 43.75 and 3.29, respectively. Patients with low NLR and high PNI had the best outcomes, potentially indicative of the intensive antitumor effects of the immune system. Moreover, patients with at least three risk factors had a significantly lower OS and RFS compared with those with two or fewer risk factors. This new nomogram based on PNI and NLR may provide an accessible and individualized prediction of survival and recurrence for HCC patients.

## Introduction

Hepatocellular carcinoma (HCC) is one of the most common cancers with the mortality rate ranking fifth among men and eighth among women (1). Removal of HCC is one of the most effective treatments; however, the high incidence of recurrence remains the leading cause of death after curative resection. As the survival of patients who received potentially curative treatment remains poor, risk factors for postoperative survival should be determined to personalize therapies and improve clinical outcomes.

Increasing evidence shows that the occurrence of systemic inflammation and nutritional disorders promote carcinogenesis by inhibiting apoptosis, promoting angiogenesis, and damaging DNA (2, 3). For example, elevated C-reactive protein (CRP) concentration is associated with lower survival in patients with various malignancies, including HCC (4). Furthermore, it is reported that the presence of an inflammatory response is pathogenic in the development of cancer-associated malnutrition, especially in patients with cirrhosis (5). The prognostic nutritional index (PNI), an overall measure of a patient’s immunonutritional condition, has been demonstrated to be an independent prognostic factor in various types of cancer, such as gastric carcinoma (6), colon cancer (7), pancreatic cancer (8), and HCC (9, 10). The previous studies have been reported that the PNI was a prognostic factor for evaluating short- and long-term outcomes after liver resection in HCC patients (9) and preoperative PNI predicts prognosis after curative hepatectomy in early Barcelona clinic liver cancer (BCLC) stage HCC (11). However, in the present study, we further explore the correlation between PNI and other series of prognostic indicators and clinical features; in addition, we aimed to combine two or more risk factors to better predict the prognosis of HCC patients after hepatectomy.

A previous study suggested that the platelet–lymphocyte ratio (PLR) may provide a reliable and individualized prediction of tumor recurrence in HCC patients after radiofrequency ablation (RFA) (12). Other inflammatory-based prognostic indices, notably neutrophil-to-lymphocyte ratio (NLR), γ-glutamyl transferase/alanine aminotransferase ratio (GGT/ALT), and the aspartate aminotransferase/platelet count ratio index (APRI), have been studied for their prognostic roles for various cancers (13–15). A previous study found that high NLR is associated with poor survival in patients with unresectable and recurrent gastric cancer (14), and a high preoperative NLR stood for poor prognostic factor in HCC patients after curative resection while some were in doubt. Preoperative NLR could predict survival better than the conventional alpha-fetoprotein (AFP) in HCC patients after curative resection (16, 17), and NLR was an accurate prognostic marker for OS and PFS of unresectable intermediate and advanced HCC patients on apatinib treatment (18). However, Chan found that NLR had no prognostic significance on OS and disease-free survival in early-stage HCC (11). This may be related to the fact that the author only selected early-stage HCC patients as the research object. Lastly, different methods are used to obtain the cutoff value in previous studies; all of this may lead to the controversial prognostic value of these indices. Therefore, the novelty of the present study compared with previous studies was that the prognostic values of PNI, NLR, APRI, GGT/ALT, and PLR were analyzed in both OS and RFS after curative hepatectomy, that the analysis included all stages of HCC, and we also verified that the combination of PNI, NLR, and other risk factors improved the prognostic value.

## Materials and Methods

### Patients and Methods

This retrospective study was approved by the Institutional Review Board of Changzhou First People’s Hospital. All study participants gave written informed consent for the use of their clinical records. A total of 243 patients who received curative surgery for HCC from January 2010 to August 2018 at the Changzhou First People’s Hospital were retrospectively reviewed from our department’s prospective surgical database. Twenty-eight patients were lost to follow-up and excluded. Exclusion criteria for this study included the following: patients with recurrent or metastatic HCC (n = 10), patients deceased from causes other than HCC (n = 4), patients with preoperative fever (n = 2), infection or systemic inflammatory diseases (n = 2), patients with Child–Pugh C grade (n = 5), patients with hematologic disorders (n = 2), and lack of an entire set of laboratory data (n = 3). In total, 215 patients were ultimately included and evaluated.

Blood samples were collected from patients within 7 days before surgery and analyzed in the same laboratory. The white blood cell, neutrophil, lymphocyte, and platelet counts were measured with the automated hematology analyzer Sysmex XT-4000i (Sysmex, Kobe, Japan). The alanine aminotransferase (ALT), aspartate aminotransferase (AST), γ-glutamyl transferase (γ-GT), and albumin (ALB) were measured with immunochemistry analyzer cobas 8000 (Roche, Rotkreuz, Switzerland). Routine assessments were also performed within 7 days before surgery, including a complete physical examination, chest X-ray, abdominal ultrasound, and computed tomography (CT) or magnetic resonance imaging (MRI). Other additional examinations, such as positron emission tomography-computed tomography (PET-CT), were carried out when necessary. The tumor staging was classified using the TNM staging system for HCC, and the TNM system was assessed using the seventh edition of the Union Internationale Contre le Cancer classification (UICC) (19). The NLR was calculated by dividing neutrophil count by lymphocyte count; the PNI was calculated using the following formula: serum albumin (g/L) + 5 × lymphocyte count (10^9^/L) (20).

### Treatment and Follow-up

The extent of hepatic resection was determined based on age, preoperative risk factors, the plasma retention rate of indocyanine green at 15 min, size, number and location of tumors, and Child–Pugh grade. Curative hepatectomy was defined as radical resection when no distant metastasis was detected, and tumor clearance was macroscopically and histologically complete. Anatomical hepatectomy included left or right hepatectomy, and left lateral sectionectomy, with sectionectomy defined as any type of complete excision of at least one segment based on Couinaud’s classification. In the sectionectomy surgeries, the resection line was made along the demarcation on the liver surface after ligation of the sectional pedicle, and the trunk of the hepatic vein was exposed on the resected surface. Non-anatomical hepatectomy was defined as tumor resection with a surgical margin of 5–10 mm unless the tumor was close to the main hepatic vein or the Glissonian pedicle. Postoperative activity time was defined as the time interval after surgery when the patient gets out of bed to perform any activity. Postoperative eating time was defined as the time interval after surgery when the patient was fed fluids. Postoperative drainage tube removal time was defined as the time interval after surgery in which the drainage tube was removed as determined by slowed drainage speed before stopping. All patients were followed carefully through outpatient examinations or telephone visits after the initial treatment. The serum alpha-fetoprotein (AFP) and liver function test were measured every 3 months, and contrast-enhanced CT or MRI was performed every 6 months. Percutaneous biopsy or selective hepatic arterial angiography was performed in patients with suspected tumor recurrence. Repeated hepatectomy, RFA, and transcatheter arterial chemoembolization (TACE) were performed for patients diagnosed with HCC recurrence. Disease-specific overall survival (OS) was defined as the interval between the time of surgery to the time of HCC-related death or the date of the last follow-up if death had not occurred. Disease-free survival (DFS) was the time interval defined as the time from surgery to radiological evidence of tumor recurrence. The start date of follow-up was the initial diagnostic date for HCC, and the end of the follow-up was the last follow-up (August 2018) or the time of death.

### Statistical Analyses

All statistical analyses were carried out using the SPSS 25.0 software (Chicago, IL, USA). A receiver operating characteristic (ROC) curve was generated to evaluate the sensitivity and specificity of the scoring systems for predicting OS. Based on the ROC curves, the cutoff values were determined by seeking the maximal sum of sensitivity and specificity. The measurement data were expressed as the mean ± standard deviation. The *c* ^2^ test or Fisher’s exact test was used, as appropriate, for categorical data. Kaplan–Meier analysis was used to analyze the survival for different groups. Differences of survival were performed using the log-rank test. The Cox proportional hazard model was employed for univariate and multivariate analyses. p values less than 0.05 (two-tailed) was considered statistically significant.

## Results

### Determine the Best Cutoff Point of PNI and NLR

We determined the best cutoff values for indices by the ROC curve analysis for predicting the 5-year OS. A PNI of 43.75 with a sensitivity of 74.5% and a specificity of 78.2% was chosen as the best cutoff point for 5-year OS, and the area under the curve (AUC) was 0.592. Similarly, the optimal cutoff value was set as 3.29 for NLR with a sensitivity of 59.5% and a specificity of 73.0%, and the AUC was 0.602. Determined in the same manner as the cutoff values of PNI and NLR, APRI, GGT/ALT and PLR did not show any discriminative value for 5-year OS (*p* > 0.05) (**Table 1**).

**Table 1:** Comparison of the AUC between inflammation-based prognostic scores.

	AUC	95% CI	*p*-value	Cutoff value
PNI	0.592	0.512–0.672	0.024	43.75
NLR	0.602	0.566–0.725	<0.001	3.29
APRI	0.448	0.367–0.528	0.198	0.14
GGT/ALT	0.466	0.386–0.547	0.409	0.62
PLR	0.475	0.395–0.555	0.538	69.17

PNI, prognostic nutritional index; NLR, neutrophil to lymphocyte ratio; APRI, aspartate aminotransferase/platelet count ratio index; GGT/ALT, gamma glutamyl transferase/alanine aminotransferase ratio; PLR, platelet–lymphocyte ratio.

### HCC Patient Characteristics and Correlation With PNI and NLR

The clinicopathologic characteristics of HCC patients with different PNI and NLR are summarized in **Table 2**. Correlation analysis of our study proved that a low PNI was significantly associated with older age, higher Child–Pugh grade, more intraoperative blood loss, worse degree of tumor differentiation, and higher recurrence rate (*p* < 0.05). Likewise, a high NLR was positively associated with a higher Child–Pugh grade and a higher recurrence rate (p < 0.01).

**Table 2:** Clinicopathological characteristics of the patients and clinicopathological correlations of inflammatory indices.

Characteristics	Overall (N = 215)	High PNI (N = 119)	Low PNI (N = 96)	*p*	Low NLR (N = 133)	High NLR (N = 82)	*p*
Age (years)	59.10 ± 10.49	57.54 ± 9.96	60.95 ± 10.86	**0.017**	58.69 ± 10.27	59.65 ± 10.87	0.521
Sex (male/female)	178/37	100/19	78/18	0.591	111/22	67/15	0.741
HBsAg positive (%)	160 (74.4%)	86(72.3%)	74(77.1%)	0.421	99(74.4%)	61(74.1%)	0.994
Child–Pugh grade (A/B)	188/27	117/2	71/25	**<0.001**	125/8	63/19	**<0.001**
Intraoperative blood loss	637.07 ± 1001.64	440.44 ± 737.65	888.33 ± 1220.20	**0.003**	613.41 ± 1019.38	677.24 ± 976.17	0.661
Tumor diameter (cm)	5.94 ± 8.60	5.23 ± 3.21	6.82 ± 12.35	0.178	5.30 ± 3.19	6.96 ± 13.31	0.169
Vascular invasion	170/45	98/23	72/22	0.432	110/23	60/22	0.095
Tumor stage (I/II/III)	149/52/14	84/27/8	65/25/6	0.421	95/31/7	54/21/7	0.765
Degree of differentiation	49/139/27	29/73/17	20/66/10	**<0.001**	25/87/21	24/52/6	0.068
Postoperative death	211/4	118/1	93/3	0.469	130/3	81/1	0.979
Recurrence	119/92	89/29	30/63	**<0.001**	82/47	3745	**0.007**

The bold values meaning statistically significant.

### Univariate and Multivariate Analyses of Prognostic Factors for OS and RFS

**Figure 1:**
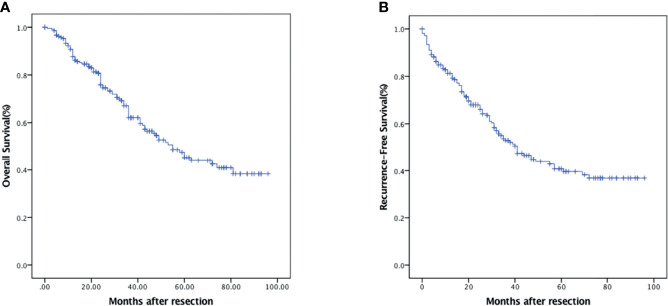
Kaplan–Meier survival analysis of HCC patients. **(A)** OS in HCC patients who underwent curative hepatectomy. **(B)** RFS in HCC patients who underwent curative hepatectomy.

The median duration of follow-up was 31 (range 3–96) months. In all, 92 patients (42.8%) developed recurrence, and 88 (40.9%) died during follow-up. The 1-, 3-, and 5-year OS were 87.7% (95% CI: 83.2%–92.2%), 62.1% (95% CI: 54.7%–69.5%), and 45.3% (95% CI: 36.7%–53.9%), respectively (**Figure 1A**). The 1-, 3-, and 5-year RFS were 81.3% (95% CI: 76.0%–86.6%), 52.9% (95% CI: 45.5%–60.3%), and 40.8% (95% CI: 32.8%–48.8%), respectively (**Figure 1B**).

The results of the Cox regression hazard model for predictors of overall survival are shown in **Table 3**. In univariate analysis, CRP, Child–Pugh grade, tumor diameter, vascular invasion, tumor stage, degree of differentiation, intraoperative transfusion, PNI, and NLR were significant predictors of OS (*p* < 0.05). A multivariate analysis of significant variables showed that tumor diameter (HR: 1.100, 95% CI: 1.011–1.197, *p* = 0.026), tumor stage (HR: 0.149, 95% CI: 0.061–0.365, *p* < 0.001), degree of differentiation (HR: 3.684, 95% CI: 1.978–6.861, *p* < 0.001), PNI (HR: 5.081, 95% CI: 2.209–11.688, *p* < 0.001), and NLR (HR: 0.510, 95% CI: 0.272–0.957, *p* = 0.036) were independently associated with OS. The outcomes of the Cox regression hazard model for predictors of OS are shown in **Table 3**.

**Table 3:** Univariate and multivariate analyses of prognostic factors for overall survival of patients.

Variable	Univariate		Multivariate	
	OS HR (95% CI)	*p*	OS HR (95% CI)	*p*
Age (years)	1.013 (0.991–1.035)	0.250		
Sex (male/female)	0.972 (0.5340–1.770)	0.926		
HBsAg positive	0.697 (0.425–1.141)	0.151		
Cirrhosis	1.205 (0.763–1.902)	0.425		
CRP (mg/L)	1.018 (1.005–1.032)	**0.007**	0.994 (0.975–1.014)	0.561
AFP (ng/mL)	1.159 (0.675–1.992)	0.592		
Child–Pugh grade (A/B)	2.587 (1.511–4.428)	**0.001**	0.700 (0.237–2.069)	0.519
Tumor diameter (cm)	1.025 (1.003–1.046)	**0.023**	1.100 (1.011–1.197)	**0.026**
Vascular invasion	0.302 (0.188–0.486)	**<0.001**	0.828 (0.395–1.737)	0.617
Tumor stage (I and II/III)	0.388 (0.199–0.757)	**0.005**	0.149 (0.061–0.365)	**<0.001**
Degree of differentiation	2.105 (1.277–3.468)	**0.004**	3.684 (1.978–6.861)	**<0.001**
Intraoperative blood loss (mL)	1.000 (1.000–1.001)	0.050		
PNI (≥43.75/<43.75)	6.176 (3.523–10.862)	**<0.001**	5.081 (2.209–11.688)	**<0.001**
NLR (≥3.29/<3.29)	0.283 (0.176–0.455)	**<0.001**	0.510 (0.272–0.957)	**0.036**

Likewise, the univariate analysis indicated that CRP, Child–Pugh grade, vascular invasion, tumor stage, degree of differentiation, PNI, and NLR were significant predictors of RFS (*p* < 0.001). A multivariate analysis of significant variables showed that vascular invasion (HR: 0.513, 95% CI: 0.277–0.952, *p* = 0.034), tumor stage (HR: 0.184, 95% CI: 0.059–0.569, *p* = 0.003), degree of differentiation (HR: 2.836, 95% CI: 1.593–5.050, *p* < 0.001), and PNI (HR: 6.530, 95% CI: 3.456–12.338, *p* < 0.001) were independently associated with RFS. The outcomes of the Cox regression hazard model for predictors of RFS are shown in **Table 4**.

**Table 4:** Univariate and multivariate analyses of prognostic factors for recurrence-free survival of patients.

Variable	Univariate		Multivariate	
	HR (95% CI)	*p*	HR (95% CI)	*p*
Age (years)	1.003 (0.984–1.023)	0.738		
Sex (male/female)	0.883 (0.507–1.535)	0.658		
HBsAg positive	1.287 (0.817–2.028)	0.276		
Cirrhosis	1.171 (0.778–1.764)	0.450		
CRP (mg/L)	1.015 (1.002–1.029)	**0.027**	0.988 (0.968–1.007)	0.218
AFP (ng/mL)	0.784 (0.483–1.272)	0.324		
Child–Pugh grade (A/B)	0.314 (0.190–0.517)	**<0.001**	0.775 (0.290–2.071)	0.611
Tumor diameter (cm)	1.015 (0.997–1.033)	0.096		
Vascular invasion	0.379 (0.244–0.589)	**<0.001**	0.513 (0.277–0.952)	**0.034**
Tumor stage (I and II/III)	0.136 (0.073–0.256)	**<0.001**	0.184 (0.059–0.569)	**0.003**
Degree of differentiation	1.404 (0.932–1.886)	**<0.001**	2.836 (1.593–5.050)	**<0.001**
Intraoperative blood loss (mL)	1.000 (1.000–1.001)	0.279		
PNI (≥43.75/<43.75)	3.846 (2.451–6.035)	**<0.001**	6.530 (3.456–12.338)	**<0.001**
NLR (≥3.29/<3.29)	0.448 (0.296–0.679)	**<0.001**	0.972 (0.435–2.170)	0.944

### Overall and Recurrence-Free Survival Curve According to NLR and PNI

**Figure 2:**
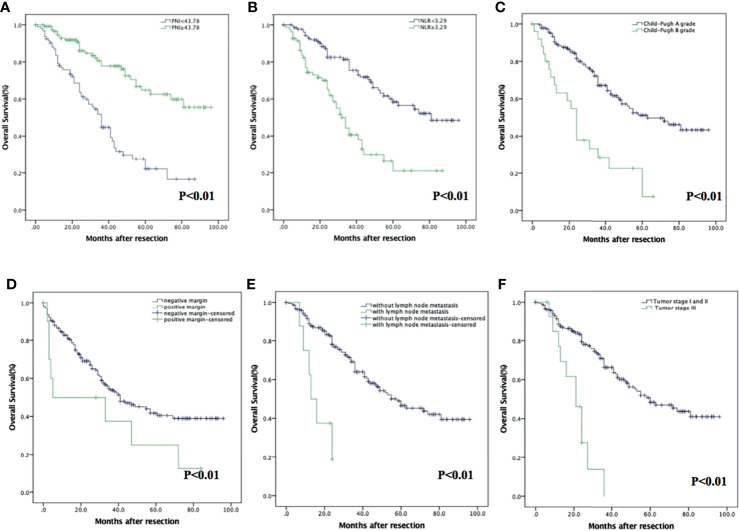
Kaplan–Meier analysis of HCC patients showing OS curves stratified according to PNI **(A)**, NLR **(B)**, Child–Pugh grade **(C)**, vascular invasion **(D)**, tumor stage **(E)**, and degree of differentiation **(F)**.

The OS in the PNI-high group were significantly higher than those in the PNI-low group (*p* < 0.001). The 1-, 3-, and 5-year OS were 94.6% (95% CI: 90.5%–98.7%), 77.8% (95% CI: 68.9%–86.6%), and 62.6% (95% CI: 50.6%–74.6%) in the PNI-high group; 79.1% (95% CI: 70.7%–87.5%), 44.8% (95% CI: 33.8%–55.8%), and 22.1% (95% CI: 10.9%–33.3%) in the PNI-low group (**Figure 2A**). The OS in the NLR-low group were significantly higher than those in the NLR-high group (*p* < 0.001). The 1-, 3-, and 5-year OS were 94.4% (95% CI: 90.3%–98.5%), 75.5% (95% CI: 67.1%–83.9%), and 58.4% (95% CI: 47.6%–69.2%) in the NLR-low group; 77.0% (95% CI: 67.6%–86.4%), 40.6% (95% CI: 28.3%–52.9%), and 26.3% (95% CI: 12.4%–40.2%) in the NLR-high group (**Figure 2B**). Expected tumor number (*p* = 0.059), other prognostic factors, such as Child–Pugh A grade (*p* < 0.001); without vascular invasion (*p* < 0.001), moderate or good differentiation (*p* = 0.006); and tumor stage I or II (*p* < 0.001) were associated with an increased OS (**Figures 2C–F**).

**Figure 3:**
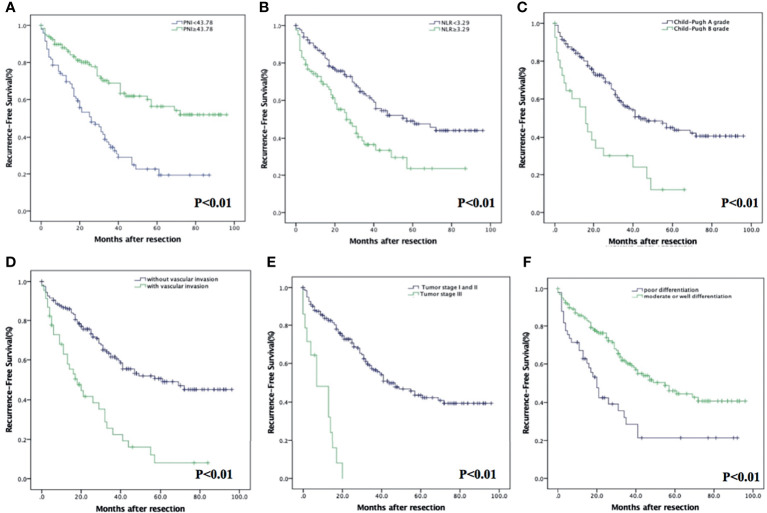
Kaplan–Meier analysis of HCC patients showing RFS curves stratified according to PNI **(A)**, NLR **(B)**, Child–Pugh grade **(C)**, vascular invasion **(D)**, tumor stage **(E)**, and degree of differentiation **(F)**.

Homoplastically, the RFS in PNI-high were significantly higher than those in the PNI-low group (*p* < 0.001). The 1-, 3-, and 5-year RFS were 87.9% (95% CI: 82.0%–93.8%), 68.9% (95% CI: 59.5%–78.3%), and 56.3% (95% CI: 45.1%–67.5%) in the PNI-high group; 73.0% (95% CI: 64.0%–82.0%), 34.6% (95% CI: 24.2%–45.0%), and 19.3% (95% CI: 8.7%–29.9%) in the PNI-low group (**Figure 3A**), respectively. The RFS in the NLR-low group were significantly higher than those in the NLR-high group (*p* < 0.001). The 1-, 3-, and 5-year RFS were 86.7% (95% CI: 80.8%–92.6%), 62.5% (95% CI: 53.5%–71.5%), and 49.2% (95% CI: 39.2%–59.2%) in the NLR-low group; 72.8% (95% CI: 63.0%–82.6%), 36.7% (95% CI: 24.5%–48.9%), and 23.5% (95% CI: 8.6%–38.4%) in the NLR-high group (**Figure 3B**), respectively. Other prognostic factors, such as Child–Pugh A grade (*p* < 0.001), without vascular invasion (*p* < 0.001), moderate or good differentiation (*p* < 0.001), and tumor stage I or II (*p* < 0.001), were associated with an increased RFS (**Figures 3C**–**F**).

### The Prognostic Value Ground on the Combination of NLR and PNI

**Figure 4:**
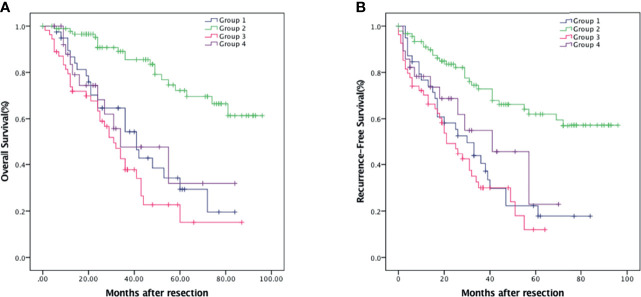
Kaplan–Meier survival analysis of HCC patients showing survival curves stratified according to the combination of NLR and PNI. OS **(A)** and RFS **(B)** curve comparing different groups. Group 1, NLR <3.29 and PNI <43.75 (n = 42); Group 2, NLR <3.29 and PNI ≥43.75 (n = 91); Group 3, NLR ≥3.29 and PNI <43.75 (n = 54); Group 4, NLR ≥3.29 and PNI ≥43.75 (n = 28).

Patients were divided into four groups to analyze the prognostic value of NLR combined with PNI: Group 1, the both low NLR and PNI group (n = 42, 19.5%); Group 2, the low NLR and high PNI group (n = 91, 42.3%); Group 3, the high NLR and low PNI group (n = 54, 25.1%); and Group 4, the both high NLR and PNI group (n = 28, 13%). Group 2 which stood for the intensive antitumor effects of the immune system had the best outcome while group 3 which represented the depression and malnutrition of the immune system had the worst outcome (*p* < 0.001). The 1-, 3-, and 5-year OS were 86.7% (95% CI: 75.7%–97.7%), 54.4% (95% CI: 37.5%–71.3%), and 29.3% (95% CI: 11.9%–46.7%) in group 1; 97.7% (95% CI: 94.6%–100%), 85.5% (95% CI: 77.1%–93.9%), and 72.2% (95% CI: 60.1%–84.4%) in group 2; 73.8% (95% CI: 62.0%–85.6%), 37.8% (95% CI: 23.7%–51.9%), and 15.1% (95% CI: 0.1%–30.4%) in group 3; 83.4% (95% CI: 68.5%–98.3%), 47.8% (95% CI: 23.7%–71.9%), and 31.9% (95% CI: 1.7%–62.1%) in group 4, respectively (**Figure 4A**). Accordingly, the 1-, 3-, and 5-year RFS were 79.3% (95% CI: 66.6%–92.0%), 40.5% (95% CI: 24.4%–56.6%), and 17.8% (95% CI: 3.5%–32.1%) in group 1; 91.0% (95% CI: 84.9%–97.1%), 72.8% (95% CI: 62.6%–83.0%), and 62.0% (95% CI: 50.2%–73.8%) in group 2; 70.2% (95% CI: 58%–82.4%), 30.0% (95% CI: 16.5%–43.5%), and 12.0% (95% CI: 1.3%–27%) in group 3; and 73.6% (95% CI: 56.5%–90.7%), 45.8% (95% CI: 20.9%–70.7%), and 22.9% (95% CI: 3.3%–49.2%) in group 4, respectively (**Figure 4B**).

### Combinations of Risk Factors Better Predict OS and RFS

**Figure 5:**
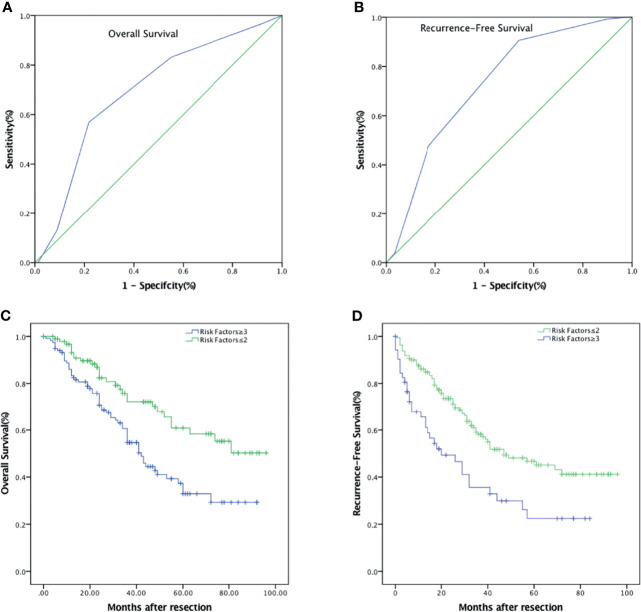
Combinations of independent risk factors better predict survival of patients with HCC after hepatic resection. **(A)** ROC of combined risk factors for the prediction of OS. **(B)** ROC of combined risk factors for the prediction of RFS. **(C)** OS curve comparing risk factors ≥3 and risk factors ≤2. **(D)** RFS curve comparing risk factors ≥3 and risk factors ≤2.

We then try to find out if the combination of independent prognostic factors could provide a better diagnostic value for OS and RFS. ROC analysis showed that for predicting OS, when the number of combined risk factors was set to >2.5, the model had the best predictive value with an AUC = 0.694 (*p* < 0.001, **Figure 5A**). The sensitivity and specificity were 56.9% and 78.2%, respectively. The OS in patients with at least three risk factors were significantly lower than those with two or fewer risk factors (*p* < 0.001, **Figure 5C**). The 1-, 3-, and 5-year OS were 83.4% (95% CI: 76.5%–90.3%), 54.7% (95% CI: 44.7%–64.7%), and 32.9% (95% CI: 21.7%–44.1%) in patients with at least three risk factors, and 93.2% (95% CI: 87.9%–98.5%), 72.2% (95% CI: 61.6%–82.8%), and 58.3% (95% CI: 45.2%–71.4%) in patients with two or less risk factors, respectively. Likewise, for RFS, the optimal cutoff value for the number of combined risk factors was >2.5. The sensitivity and specificity of this model were 90.6% and 46.0%, respectively (AUC = 0.732, p < 0.001, **Figure 5B**). The RFS in patients with at least three risk factors were significantly lower than those with two or fewer risk factors (*p* = 0.001, **Figure 5D**). The 1-, 3-, and 5-year RFS were 65.6% (95% CI: 52.5%–78.7%), 35.6% (95% CI: 21.5%–49.7%), and 22.4% (95% CI: 9.3%–35.5%) in patients with at least three risk factors, and 86.1% (95% CI: 80.8%–91.4%), 58.1% (95% CI: 49.5%–66.7%), and 45.1% (95% CI: 35.7%–54.5%) in patients with two or less risk factors, respectively.

## Discussion

Surgical resection is the primary method for the curative treatment of HCC, but the major complication of this procedure is tumor recurrence. Factors such as AFP, TNM staging, and liver reserve function can be used to predict the survival of HCC patients after the operation and the risk of tumor recurrence. However, these traditional predictive indices have practical limitations in predicting the prognosis of HCC, such as the high negative detection rate (over 30%) of serum AFP and the hysteresis of TNM (21). Therefore, the identification of reliable and simple prognostic biomarkers is essential for identifying patients with potentially poor prognosis prognoses.

The importance of host inflammatory responses points to the utility of inflammatory indices for predicting clinical outcomes in patients with cancers. The most common causes of HCC patients, especially in China, are related to chronic viral hepatitis infections (B and C). Several inflammatory-based prognostic indices (NLR, PNI, PLR, and GGT/ALT) and liver fibrosis predictor (APRI) were examined in this study. The PNI was initially designed to assess the risk of surgical complications and mortality in patients undergoing gastrointestinal tract surgery (22). Increasing studies suggested that preoperative PNI could predict the overall survival of HCC patients undergoing surgery or targeted therapy (9, 10, 23). Our study is somewhat different from previous studies. Firstly, instead of only selecting BCLC stage 0/A primary HCC patients as the research object, all patients including some HCC patients with clinically middle to advanced stage who received curative surgery were retrospectively reviewed in the present study. It is well known that HCC progression could lead to liver function damage, biliary tract infection, and nutritional deficiency; these may better confirm the prognostic values of PNI and NLR. Secondly, different methods are used to obtain the cutoff value in the previous study; Chan used the mean or median of PNI (45) and NLR (5) as the cutoff value, and they found that NLR had no prognostic significance on OS and disease-free survival (11). The best cutoff value in each institute can be established by the receiver operating characteristic curve, and the best cutoff value for balancing sensitivity and specificity was determined by Youden index = (sensitivity + specificity-1) (16). We use this method in the present study, and the best cutoff value of PNI was 43.75 and presented a sensitivity of 74.5% and a specificity of 78.2%; similarly, the optimal cutoff value was set as 3.29 for NLR with a sensitivity of 59.5% and a specificity of 73.0%.

In the present study, we collected and analyzed the data of HCC patients at our institution seeking to identify more definitive correlations. After excluding 28 patients with preoperative underlying diseases or postoperative complications, our study determined that a preoperative PNI greater than 43.78 was an independent prognostic factor for favorable OS and RFS. Lymphopenia and/or hypoalbuminemia result in low survival, and we speculate that several immunological factors are involved and lymphocytes play an important role in the eradication of the formation and development of tumors (24, 25). CD8+ and CD4+ lymphocytes can induce antitumor immunity, and therefore, HCC characterized by inflammatory cell infiltration would have a better prognosis (26). Previous studies have demonstrated that age is inversely correlated with liver regeneration in humans (27). In our study, the PNI-low group, composed of older patients, had worse survival than the PNI-high group after surgery. Therefore, good nutrition and immune status before surgery are important factors for the improvement of postoperative survival, particularly in elderly patients.

Several studies have shown that high preoperative NLR is a predictor of negative prognosis for patients receiving liver transplantation or surgical treatment (28). However, some researchers doubt the utility of preoperative NLR as an independent prognostic factor in HCC patients, supposing that it is only an indicator of the patient’s overall inflammation status (11). In the present study, the proportion of hepatitis B-positive patients (74.4%) was higher than in previous studies; these may lead to poor Child–Pugh grade and poor OS. In addition, NLR was associated with inflammatory activity, and it also upregulated in HCC patients with HBV infection (16). Our results suggested that high NLR (≥3.29), indicating a relative increase in neutrophils or decrease in lymphocytes, is an independent adverse prognostic factor for OS. Although the present study could not demonstrate any association between the NLR and RFS, this may be related to our limited number of patients. Intriguingly, the PNI and NLR combination can enhance the accuracy of PNI in predicting RFS.

In the present study, we also verified that the combination of PNI, NLR, and other risk factors improved the prognostic value. Risk factors for OS include serum albumin concentration, tumor diameter, tumor stage III, and poor differentiation, and risk factors for RFS include vascular invasion, tumor stage III, and poor differentiation. We found that after combining risk factors, the model had a better predictive value with a higher AUC when the number of risk factors was greater than 2. Appropriate plans can be put in place to prevent recurrence and achieve long time survival (29). Also, survival in patients with a low PNI and high NLR may be improved by nutritional and anti-infection therapy (30). In addition, intensive postoperative follow-up or postoperative adjuvant therapy could be applied for patients with more than two risk factors.

In conclusion, our study has demonstrated that PNI and NLR are independent prognostic factors for the OS of all clinical stages of HCC patients undergoing hepatectomy. Moreover, PNI is a reliable predictor of RFS in postoperative HCC patients. Importantly, monitoring combined risk factors can increase accuracy for predicting the OS and RFS in HCC patients.

## Data Availability Statement

The original contributions presented in the study are included in the article/**Supplementary Material**. Further inquiries can be directed to the corresponding authors.

## Ethics Statement

Written informed consent was obtained from the individual(s) for the publication of any potentially identifiable images or data included in this article.

## Author Contributions

Conception and design: ZQ, Y-FD, and J-WF. Data collection: ZQ and J-WF. Writing of the article: ZQ, Y-FD, J-WF, Y-xC, JC, L-qS, NR, and Y-jL. Critical revision of the article: Y-jL, NR, and X-zH. Final approval of the article: ZQ, Y-jL, J-WF, Y-xC, JC, L-qS, NR, Y-FD, and X-zH. Statistical analysis: ZQ, Y-FD, J-WF, and Y-jL. Obtained funding: ZQ and Y-jL. Overall responsibility: Y-FD. All authors contributed to the article and approved the submitted version.

## Funding

The research was supported by the National Natural Science Foundation of China (no. 81971504) and Scientific and Technological Projects for Young Talents, Changzhou Health and Family Planning Commission (QN201809). Changzhou Society Development Funding (CE20205038), The lifting Project of Young Scientific and technological talents in Changzhou (2021).

## Conflict of Interest

The authors declare that the research was conducted in the absence of any commercial or financial relationships that could be construed as a potential conflict of interest.

## Publisher’s Note

All claims expressed in this article are solely those of the authors and do not necessarily represent those of their affiliated organizations, or those of the publisher, the editors and the reviewers. Any product that may be evaluated in this article, or claim that may be made by its manufacturer, is not guaranteed or endorsed by the publisher.
